# Is TGF-β Associated with Cytokines and Other Biochemical or Clinical Risk Parameters in Early-Onset CAD Patients?

**DOI:** 10.3390/biomedicines13081840

**Published:** 2025-07-29

**Authors:** Bartosz Rakoczy, Violetta Dziedziejko, Krzysztof Safranow, Monika Rac

**Affiliations:** 1 Department of Clinical Radiology, University Hospital of Karol Marcinkowski, 26 Zyty St., 65-046 Zielona Góra, Poland; b_rakoczy@icloud.com; 2 Department of Biochemistry, Pomeranian Medical University, Powstańców Wielkopolskich 72, 70-111 Szczecin, Poland; viola@pum.edu.pl (V.D.); chrissaf@mp.pl (K.S.)

**Keywords:** TGF-β, CAD, atherosclerosis risk factor

## Abstract

**Background:** TGF-β is an immunosuppressive cytokine. Its signaling pathway plays a role in anti-inflammatory responses. Coronary artery disease (CAD) is a clinical consequence of atherosclerosis, which manifests as chronic inflammation and involves platelet mediators, including TGF-β. The aim of this study is to validate the diagnostic utility of TGF-β levels in relation to classical and molecular risk factors for CAD. **Methods:** The study group included 25 women and 75 men, all aged up to 55 and 50 years, respectively, who had been diagnosed with early-onset CAD. Fasting blood samples were taken to measure plasma levels of TGF-β, sCD36, PCSK9, TNF, VEGF, IL-6, and E-selectin using the ELISA method. Furthermore, a full lipid profile, apolipoproteins (Lp(a), ApoA1, and ApoB), C-reactive protein (hsCRP), and blood morphology were analyzed at the Central Hospital Laboratory. A physical examination was also performed. **Results:** Positive associations were observed between TGF-β concentration and TNF, platelet count, PTC, and triglyceride levels. TNF and platelet concentration were significant independent predictors of increased plasma TGF-β levels. None of the clinical parameters showed statistically significant associations with plasma TGF-β concentration. **Conclusions:** Our research has demonstrated that TGF-β levels, including circulating TNF, triglycerides, and platelets, are linked to specific biochemical risk factors in early-onset CAD cases.

## 1. Introduction

As a multifunctional cytokine, transforming growth factor (TGF-β) is expressed in many cell types and tissues. It mediates signaling functions, particularly involved in tissue-specific control of cell proliferation, differentiation, and cell and tissue-specific motility [1]. Platelets are the most abundant source of TGF-β among all tissues. It is an immunosuppressive cytokine [2]. The Introduction Section of this study intends to help readers better understand the role of TGF and other cytokines in inflammation during CAD.

**Figure 1:**
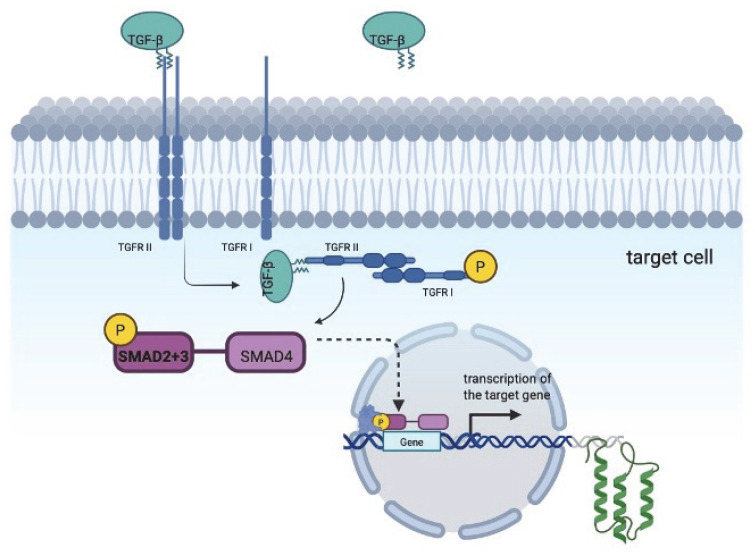
TGF-β regulatory cascade.

The human gene of the TGF-β family encodes secreted cytokines. These proteins are synthesized as dimeric precursors, which are cleaved during processing in the secretory pathway [3]. After maturation, the two parts of TGF-β remain associated, forming the latent form of the ligand. It lacks biological activity in the absence of further processing. The latent complex form of TGF-β contains critical amino acids within its C-terminal dimer, which are essential for interaction with signaling receptors. These amino acids are covered by the N-terminal part of the pro-peptide, known as the latency-associated peptide (LAP). Both latent and mature TGF-β ligands predominantly exist as homodimers. Heteromeric ligands have different biological functions compared to their homodimeric counterparts [4]. The activation of latent TGF-β mediates two mechanisms: an integrin-based activation mechanism and the protease-dependent mechanism [5,6]. Upon activation, mature TGF-β directly binds to plasma membrane receptors, initiating the signal transduction cascade. TGF-β receptors have catalytic activity and act as their own ATP-dependent protein kinases. Alternative TGF-β signaling pathways involve Ras and Rho GTPase proteins, as well as mitogen-activated protein (MAP) kinases [7]. Some cell surface proteins act as coreceptors by either inhibiting or facilitating signaling via TGF-β receptors [8]. After ligand binding, the receptor and TGF-β complex are internalized into the cell via an endocytic process [9]. Following internalization, TGF-β receptors are degraded in lysosomes or they are recycled to the plasma membrane, prolonging TGF-β signaling [10]. Figure 1 shows the TGF-β regulatory cascade. TGF-β is a ligand for two membrane receptors, namely, TGF-β RI and RII, which become activated when they bind to the ligand. The final effect of ligand binding depends on the ratio of receptors. TGF-β has high binding specificity for the type II receptor, with the cytokine initially binding to this receptor. It then binds to the type I receptor, resulting in the formation of a complex comprising two TGF-βRII and two TGF-βRI molecules. Upon association, the type II receptor phosphorylates the serine and threonine residues of this complex. Following the association of TGF-β with its receptor, the classic activation pathway is associated with the phosphorylation of SMAD2 and SMAD3, leading to their association with SMAD4. This complex then enters the nucleus and regulates gene transcription. SMAD can be phosphorylated by ERK kinases, which inhibits SMAD2/3 phosphorylation and, consequently, the TGF-β-dependent signal. High expression of the type II receptor has vasoprotective, anti-inflammatory, and anti-atherosclerotic effects. Conversely, overexpression of type I receptors promotes pathological phenomena such as fibrosis [11,12]. TGF-β-dependent processes are of particular interest in the context of fibrosis and neovascularization of ischemic myocardium. TGF-β blocks the expression of cyclins D2 and E, as well as CDK4 and c-Myc, in T lymphocytes. It also inhibits the differentiation of T1 and T2 lymphocytes by suppressing the expression of transcription factors: T-bet and Stat-4 in T1 lymphocytes and GATA-3 and Stat-6 in T2 lymphocytes [13].

**Figure 2:**
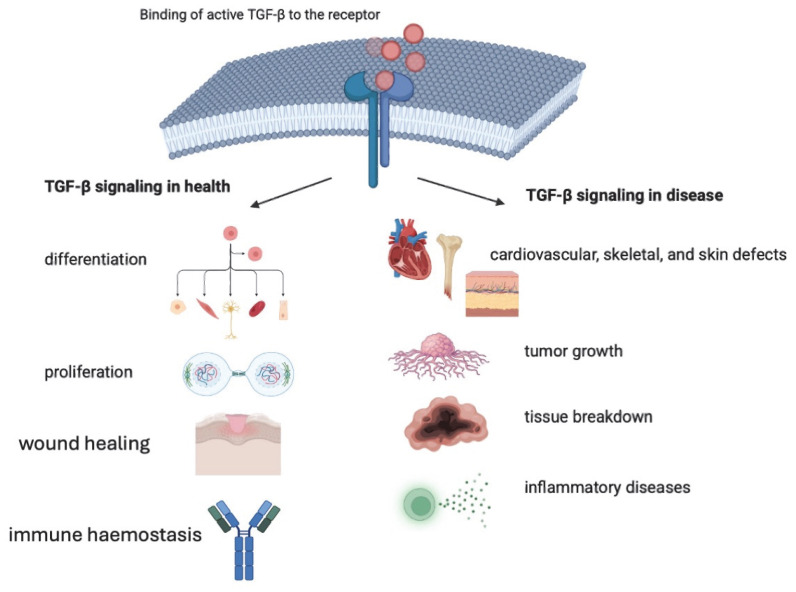
TGF-β signaling in different metabolic processes.

TGF-β creates a signaling network of signals during embryonic and neonatal development, during tissue and immune homeostasis in adult health, and during specific diseases and their progression [14]. Figure 2 shows the involvement of the TGF-β signaling pathway in different metabolic processes. TGF-β signaling is widespread, and pathway regulation at multiple levels is essential for it to function physiologically. Dysregulation of the TGF-β family of signaling results in weakened or, more commonly, enhanced TGF-β signaling [15]. TGF-β regulates cell proliferation and migration in the endothelium and recruits macrophages, which upregulate TGF-β expression. Angiogenesis in the TGF-β-induced mechanism results in vascular endothelial growth factor (VEGF) induction in epithelial cells [16]. TGF-β can impair IL expression, but, at the same time, inhibits macrophage cytotoxicity by downregulating a few cytotoxic factors, as well as TNF-α. TGF-β has chemotactic activity. In vascular injury, TGF-β promotes infiltration of inflammatory cells into the EMC and mobilizes MSCs in the peripheral blood. The aim is to deliver cells to the injured sites for vascular repair. In that way, TGF-β induces the expression of epicardial cells after myocardial injury, which then migrate to the injured myocardium [17,18].

**Figure 3:**
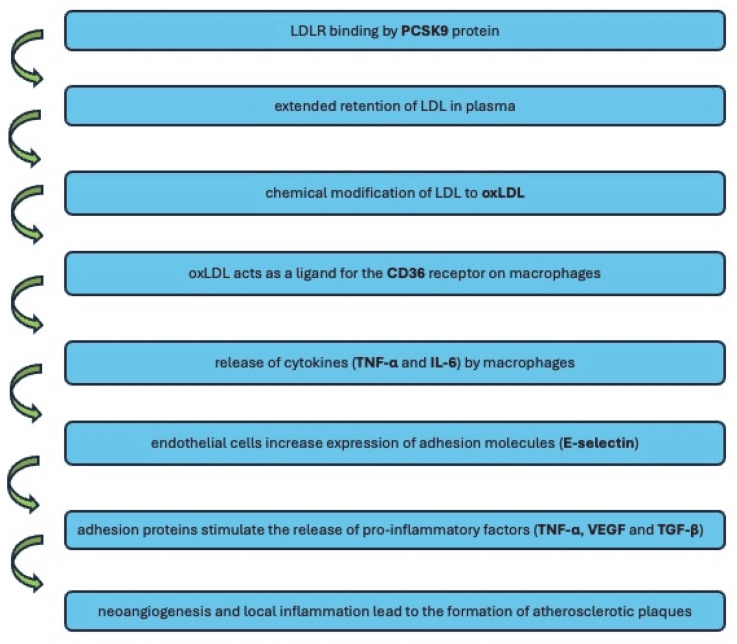
Associative bonds between the studied factors, lipid metabolism, and atherosclerotic plaque formation.

Atherosclerosis is a multicomplex pathological process that leads to life-threatening conditions due to inflammation and vascular abnormalities. During this long process, secreted pathophysiological factors influence the disease outcome. In the atherosclerotic plaque, circulating immune cells participate in lipid infiltration and endothelial injury. In our study, we examined the plasma levels of selected pro-inflammatory factors and receptors associated with lipid metabolism. Associative bonds between the studied factors, lipid metabolism, and atherosclerotic plaque formation are shown in Figure 3. PCSK9 (proprotein convertase subtilisin/kexin type 9) is an enzyme that binds to LDL receptors (LDLRs), which are located on the surface of liver cells. This leads to the degradation of LDLRs, thereby reducing the liver’s ability to remove LDL cholesterol from the blood. The extended retention of LDL in the plasma promotes its chemical modification, including oxidation [19]. Macrophages absorb oxLDL through endocytosis using scavenger receptors, such as CD36. The expression of these receptors is not reversibly regulated, resulting in uncontrolled lipid accumulation and the formation of foam cells. The uptake of oxLDL activates macrophages [20], which secrete inflammatory cytokines, including IL-6 and TNF-α. TNF-α is a cytokine with pleiotropic pro-inflammatory effects. Local inflammation triggers a systemic inflammatory response, mainly through IL-6, which stimulates the liver to synthesize acute-phase proteins, including C-reactive protein (CRP). This results in endothelial dysfunction, including increased endothelial cell permeability and the stimulation of adhesion molecule expression on their surface. All these processes contribute to the further recruitment of inflammatory cells into the atherosclerotic plaque [21]. E-selectin is an adhesion molecule whose expression on inflamed endothelial cells is a response to pro-inflammatory cytokines [22]. The increased production of adhesive proteins facilitates the penetration of macrophages, leukocytes, and platelets into the inner layer, where they release further pro-inflammatory factors, including the cytokine TNF-α, vascular endothelial growth factor (VEGF), and growth inhibitors, such as TGF-β (transforming growth factor beta). VEGF stimulates smooth muscle cells to penetrate from the inner layer into the intima, and it also stimulates the penetration of arterioles that supply the vessel wall (vasa vasorum) into the atherosclerotic plaque. This process of neoangiogenesis is closely related to the progression of atherosclerotic lesions [23]. The chronic inflammatory process, which is central to the development of atherosclerosis, involves both non-specific response mechanisms, which depend mainly on monocytes/macrophages, and specific response mechanisms, which are controlled by T lymphocytes. As antigen-presenting cells, macrophages can present foreign antigens to T lymphocytes, thereby linking the non-specific and specific immune responses. Most immune cells secrete TGF-β1 [24].

Recently, blood biomarker measurement has become an essential non-invasive method for monitoring CAD progression. However, these biomarkers are still less studied. CAD is considered a clinical consequence of atherosclerosis, which manifests as a chronic inflammatory condition and leads to the release of platelet mediators, including TGF-β. Research findings have not yet confirmed whether the TGF-β pathway inhibits atherosclerosis or promotes it. The aim of this study is to validate the diagnostic utility of TGF-β levels in relation to classical and molecular risk factors for early-onset CAD.

## 2. Materials and Methods

### 2.1. Study Group Characteristic

**Figure 4:**
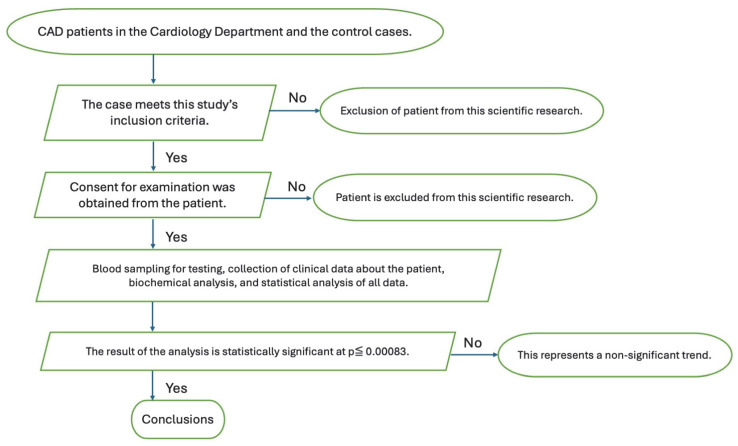
Diagram illustrating the research procedure.

The study group consisted of 25 women and 75 men, aged up to 55 and 50 years, respectively. The age of the patients was an important inclusion criterion. Several definitions of early-onset CAD have been applied in the literature [25,26], including the international society’s definitions of early-onset CAD and premature CAD. The term “premature CAD” has been reserved for CAD onset <55 years in males and <65 years in females [27,28]. Cases of early-onset CAD are defined by different authors depending on the objectives of their study [29]. In this study, we selected patients with diagnosed CAD in women up to 55 years of age and men up to 50. The age of the patients was an important inclusion criterion. It is known that only cases with strong nonclassical atherosclerotic risk factors develop early-onset CAD. Risk factors such as inflammation, the environment, thrombosis, and genetics play an important role in atherosclerosis, which leads to CAD in younger individuals. Patients were also required to have one of three conditions: documented myocardial infarction, angiographically documented coronary stenosis (more than 50% of the left main coronary artery or more than 70% of the branches), or myocardial revascularization by PTCA or CABG. Each case included in this study was 30 days after a potential cardiological procedure. We recruited consecutive and clinically stable patients receiving medical treatment for cardiac reasons. The exclusion criteria were heart failure (NYHA ≥ II), acute coronary syndrome, and other heart disease, type 1 diabetes, thyroid disease, renal disease (creatinine > 3 mg/dL), liver failure, rheumatoid arthritis, and cancer. All subjects were from the Cardiology Department in Szczecin’s Regional Hospital. The control group for the TBG level consisted of 50 healthy individuals who did not have CAD but were of the same age and sex proportions as the early-onset CAD group. These individuals were randomly selected from those who attended the Occupational Medicine Clinic for periodic medical check-ups. The workflow in Figure 4 visually demonstrates this study.

Our research was exclusively in vivo and conducted on patients with no use of in vitro methods or cell cultures. The Bioethics Committee of the Pomeranian Medical University approved this study in resolution no. BN-001/162/04, dated 6 November 2017. This study was performed in accordance with the Declaration of Helsinki. Informed consent was obtained from all patients participating in this study. Complete study documentation for each patient is kept at the Department of Biochemistry, PUM.

### 2.2. Diagnostic Tests

#### 2.2.1. Physical Examination

Morphometric measurements (weight, height, hip and waist circumference) were taken in all patients, then BMI and WHR ratios were calculated. Blood pressure was measured using the RR method, and mean arterial pressure was calculated. In addition, a medical history was collected from each patient. Table 1 presents the patients’ clinical and biochemical parameters.

#### 2.2.2. Biochemistry

Blood samples were collected from fasting forearm veins at the Hospital Central Laboratory, where standard automated methods were employed to determine blood counts, lipid parameters, and glucose levels.

#### 2.2.3. Testing Plasma TGF-β and Other Protein Levels by the ELISA Method

Fasting blood samples were taken to measure the plasma protein levels of TGF-β, sCD36, PCSK9, TNF, VEGF, IL-6, and E-selectin using an ELISA method with an immunoenzymatic assay kit (EIAab, Wuhan EIAab Science Co., Ltd., Wuhan, China). The blood was collected in an EDTA tube and then centrifuged at 4000× *g* for 10 min. The plasma samples were stored at −80 °C until analysis. Sample preparation for measuring protein concentrations was carried out using our own corrected ELISA assay methodology. It was developed based on the manufacturer’s protocol for the ELISA assay and our previously published methodology. Full details of the measurement methodology were published previously. Therefore, we refer you to our publications [23,30]. Protein concentrations were determined using an automated ELX 808IU Microplate Reader (Bio-Tek Instruments Inc., Winooski, VT, USA), which was calibrated with recombinant human protein within the appropriate range. The limit detection of TGF-β was 4.61 pg/mL, intra-assay precision (CV%) was 2.5–2.9%, and inter-assay precision was 6.4–9.1%.

### 2.3. Statistical Methods

Statistical analyses were calculated with Statistica 13 software. In the Shapiro–Wilk test, in most cases, the distributions of the measurable clinical parameters were significantly different from normal. In the next step, non-parametric tests were used for calculations, such as the Mann–Whitney U test for pairwise comparison. To assess the significance of correlations between quantitative variables, Spearman’s rank correlation coefficient was used. To determine independent predictors of TGF-β concentration, multiple linear regression analysis was performed. *p*-values < 0.05 are statistically significant, but in our study, we also applied the classical Bonferroni correction. In the analysis, a total of 60 statistical associations were analyzed. Therefore, we applied the Bonferroni-corrected threshold *p*-value in this manuscript, which is 0.00083 (0.05/60). This means that only results with a *p*-value of less than 0.00083 are considered statistically significant. Applying this correction significantly increases the statistical rigor of the associations between variables and correlations.

## 3. Results

Table 2 shows the results of an analysis comparing TGF-β concentrations in the early-onset CAD patients and the control group. Both groups were also divided by gender. No statistically significant differences in TGF-β concentration were observed in any of the three subgroups.

Table 3 shows the correlations between the clinical quantitative parameters of the patients with early-onset CAD and the concentration of circulating TGF-β. Using the standard criterion of significance (*p* = 0.05), the BMI ratio was found to be positively correlated with TGF-β levels. However, the positive correlation between TGF-β concentration and BMI is no longer statistically significant when the Bonferroni correction is applied.

Table 4 shows the associations between clinical parameters, including treatment, and circulating TGF-β concentration in the early-onset CAD patients. There were no statistically significant associations between plasma TGF-β concentration and any of the clinical parameters, except for nitrate treatment (*p* < 0.05).

Table 5 shows the correlations between biochemical parameters and plasma TGF-β concentration. Using the standard significance criterion of *p* = 0.05, positive correlations were found between TGF-β and TNF and VEGF factors, as well as with components of the lipid profile: total cholesterol, LDL, triglyceride levels, and glucose concentration. In terms of morphology, positive correlations were found between TGF-β and platelet count, white blood cell count, and platelet hematocrit (PTC), the latter of which determines the ratio of platelet mass to total blood volume. Therefore, the test shows the percentage of platelets in the blood. The only negative correlation observed was between plasma TGF-β concentration and the plasma-soluble CD36 receptor. Most importantly, after Bonferroni correction, we still observed positive associations between TGF-β concentration and TNF, platelet count, PTC, and triglyceride levels, and these associations did not lose statistical significance. We highlighted this data in green in the table. Linear regression analysis revealed that the significant independent predictors of increased plasma TGF-β levels were increased TNF and platelet concentration. However, platelet concentration lost significance after Bonferroni correction (see Table 6).

## 4. Discussion

For the cardiovascular system to function physiologically, optimal vascular function and structure are essential. Vascular remodeling involves changes to the structure of blood vessels, including their cellular and molecular composition. The molecular pathways underlying these processes include growth factors, such as VEGF, inflammatory cytokines, such as IL6 and TNFα, and MAPK and TGF-β/Smad signaling [31]. Ox-LDL stimulation is a key signal in the macrophage activation pathway, promoting CD36 expression, activating TGF-β, and inhibiting IL-6. It reduces the inflammatory response and promotes the formation of the fibrous cap of the plaque. On the other hand, the production of VEGF and TNF leads to inflammation and lipid accumulation, aggravating the inflammatory response of macrophages and plaque formation [32]. TGF-β stimulates the chemotaxis of macrophages and fibroblasts. It also increases extracellular matrix synthesis, making it an important mediator of inflammation and a key player in processes leading to CAD [33]. In addition, several studies have revealed that microRNAs can play a regulatory role in atherosclerosis by impacting the TGF-β signaling pathway, acting as either suppressors or promoters [34]. Cardiovascular diseases caused by inflammatory reactions, such as atherosclerosis, are linked to the deregulation of TGF-β signaling [35,36]. Therefore, in our study, we examined the plasma levels of selected pro-inflammatory factors and receptors associated with lipid metabolism. We found that TNF strongly correlates with increased TGF-β concentration and is an independent predictor of high TGF-β concentration in plasma. A positive correlation with VEGF and a negative correlation with the sCD36 receptor are logical consequences of the inflammatory process in the vessels of patients with early-onset CAD. In our previous studies, we demonstrated that circulating VEGF is associated with an increased risk of atherosclerosis [37]. However, sCD36 plasma concentrations were significantly positively correlated with vascular protectors such as ApoA1 and HDL-cholesterol [38]. Others [39] also observed a positive correlation between TGF-β and TNF-α, which could be attributed to balanced expression of anti- or pro-inflammatory cytokines, characteristic of chronic inflammation in the arterial wall with atherosclerosis. The upregulation of IL-6 and TNF-α in patients with CAD is associated with hypomethylation of the IL-6 and TNF-α genes, which could serve as a potential biomarker for predicting early-onset CAD risk [40]. PCSK9 has been identified as a protein associated with both CAD and hypercholesterolemia. A high level of it in plasma is a major risk factor for the development of atherosclerosis [41]. This study examined the relationship between IL6 and PCSK9. It also examined the relationship between IL6 and TGF-β. However, no associations were found.

In the study by Ahmadi et al., soluble TGF-β levels were lower in control cases than in CAD patients. Significant correlations were observed between active TGF-β and pro-inflammatory platelet markers, such as CRP and P-selectin, in CAD patients. This pro-inflammatory state is involved in the development and progression of CAD. In contrast, direct correlations between pro-inflammatory markers and active TGF-β were almost absent in the platelets of healthy individuals [42]. In a cited study, the platelet level of TGF-β1 (latent/mature) was examined in CAD patients. Platelet-borne and soluble TGF-β1, both mature/active and latent forms, were also examined with Western blotting. In our study, we measured the plasma protein levels of TGF-β with an ELISA method. Additionally, other researchers revealed that serum levels of TGF-β and IL-6 significantly increased and decreased, respectively, in a group with CAD and hypertension compared to the control group. Serum levels of TGF-β were also higher in females than in males in the control group [43]. The exclusion criteria in the cited study were not the same as those considered in our study. It is worth noting that all CAD patients in the cited study who had been referred for coronary angiography had a history of CCU admission, or there was evidence of myocardial ischemia. We recruited consecutive and clinically stable patients. Each case included in our study was assessed 30 days after a potential cardiological procedure. The exclusion criteria were heart failure (NYHA ≥ II) and acute coronary syndrome. This may explain the differences between our findings and those cited. Other authors [44] have also demonstrated significantly higher TGF-β expression on platelets in patients with acute cardiovascular disease compared to those with stable coronary artery disease. This increase was also associated with mortality and major coronary events in patients. During the development of atherosclerosis, high levels of TGF-β expression were found in some human tissues, such as smooth muscle cells (SMCs), endothelial cells, and macrophages [45]. However, this correlation was less evident in the study by other authors [46]. In our study, there was no significant difference in TGF-β concentrations between the early-onset CAD group and the control group. This may be because blood samples were collected from early-onset CAD patients at least 30 days after acute coronary syndrome, by which time many parameters had normalized. In the post-infarction myocardium, there appears to be an overproduction of TGF-β-related genes, which play a role in repair processes that counteract adverse remodeling and fibrosis of the heart muscle [47]. Consequently, TGF-β concentrations are highest immediately after a heart attack and lower during the stabilization phase. This may explain why there was no difference in TGF-β concentrations between patients with CAD and healthy individuals in our study. However, within the early-onset CAD group, we found a clear positive correlation between TGF-β and blood platelets, as well as platelet hematocrit. We used these data to create a regression model. Platelets contain growth factors that enhance tissue repair mechanisms, including TGF-β. Therefore, autologous platelet-rich plasma is used to significantly improve injury treatment [48].

In atherosclerosis, TGF-β contributes to endothelial dysfunction, which is an early stage of disease development. It also influences key factors such as vascular smooth muscle cell function, macrophage cholesterol regulation, and plaque stability [49]. In our current study, we observed a positive correlation between TGF-β and total cholesterol, LDL fractions, and circulating triacylglycerol (TG) concentrations. A high TG plasma concentration is currently considered a major risk factor for heart attack. This positive correlation remained despite the Bonferroni correction.

The limitation of this cross-sectional study is that it cannot draw causal conclusions by its very nature. Therefore, it is difficult to determine whether TGF-β is the cause, risk factor, or result of other factors, such as elevated platelets or triglycerides. Although statistical significance was demonstrated, confounding factors need to be considered. These include factors that influence the release of TGF-beta from complexes, disturbances in the expression and regulation of TGF-beta, and its interactions with other signaling pathways. These factors may lead to misinterpretation of the results. The inflammatory cascade can affect downstream products and mediators of inflammation. Due to the specific nature of our study, however, these factors were not considered.

## 5. Conclusions

There is considerable debate about whether serum TGF-β levels are associated with long-term major adverse cardiovascular events in patients with coronary artery disease. Our research has shown that these levels are closely associated with certain biochemical risk factors in patients with early-onset CAD. These include circulating TNF, triglycerides, and platelets. TNF is a pro-inflammatory cytokine. A high TG level is a recognized risk factor for heart attack, and a high platelet count is a pro-thrombotic factor.

## Figures and Tables

**Table 1:** CAD patients’ parameters.

Parameter in CAD (n = 100)	Quantity
Age of patient (years)	49.9 ± 5.91
Gender (% males)	75%
Past MI	70%
After first MI (years)	44.0 ± 5.6
Hypertension presence	66%
After hypertension diagnosis (years)	42.6 ± 8.6
Diabetes type 2	13%
Cigarette smokers	89%
Years smoking	18.9 ± 9.8
CABG	37%
PTCA	71%
Anti-platelet drugs (Aspirin)	90%
Statins	96%
Calcium channel blockers	18%
Beta-blockers	88%
Diuretics	31%
ACEI	80%
ARB	17%
Heart rate (1/min)	70.7 ± 12.1
Systolic BP (mmHg)	127 ± 14.2
Diastolic BP (mmHg)	77.0 ± 9.01
MAP (mmHg)	93.8 ± 9.35
Weight (kg)	83.4 ± 17.0
BMI (kg/m^2^)	28.1 ± 3.98
WHR	0.96 ± 0.09
Waist (cm)	98.3 ± 12.5
Hip (cm)	103 ± 9
TGF-β (ng/mL)	31.45 ± 0.74
TNF-α (pg/mL)	1.33 ± 0.36
VEGF (pg/mL)	236 ± 17.2
IL-6 (pg/mL)	1.69 ± 0.77
sCD36 (µg/mL)	15.8 ±12.9
PCSK9 (ng/mL)	358 ±10.7
E-selectin (ng/mL)	35.58 ± 1.21
Platelets (G/L)	218 ± 44.6
Hemoglobin (g/dL)	14.8 ± 1.14
WBC (G/L)	6.80 ± 0.22
PTC (%)	0.23 ± 0.01
hsCRP (mg/L)	1.82 ± 2.7
Glucose (mg/dL)	107 ± 24.8
Triacylglycerols (mg/dL)	136 ± 57.1
LDL-cholesterol (mg/dL)	102 ± 36.2
HDL-cholesterol (mg/dL)	48.4 ± 11.5
Total cholesterol (mg/dL)	173 ± 40.4
ApoB/ApoA1	0.53 ± 0.15
ApoB (mg/dL)	74.0 ± 2.25
ApoA1 (mg/dL)	154 ± 38.4
Lp(a) (mg/dL)	40.3 ± 49.3

MI—myocardial infarction, CABG—coronary artery bypass grafting, PTCA—percutaneous transluminal coronary angioplasty, ACEI—angiotensin 1 converting, ARB—angiotensin receptor blockers, MAP—mean arterial pressure, BMI—body mass index, WHR—waist-to-hip ratio, TGF-β—transforming growth factor beta, TNF-α—tumor necrosis factor α, VEGF—vascular endothelial growth factor, IL-6—interleukin 6, sCD36—soluble CD36 receptor protein, PCSK9—proprotein convertase subtilisin/kexin type 9, WBC—white blood cell, PTC—platelet hematocrit, hsCRP—high-sensitivity C-reactive protein, ApoB—apolipoprotein B, ApoA—apolipoprotein A, Lp(a)—lipoprotein (a).

**Table 2:** The associations between early-onset CAD patients and the control group in circulating TGF-β concentration (ng/mL).

Group	Mean ± Sem	*p*-Value
CAD	31.45 ± 0.74	0.68
control	31.14 ± 0.74	
CAD females	30.81 ± 1.25	0.70
CAD males	31.92 ± 0.91	
control females	31.16 ± 1.70	0.83
control males	30.70 ± 1.07	

**Table 3:** The correlations between circulating TGF-β concentration (ng/mL) and quantitative clinical parameters of early-onset CAD patients.

Parameters	Correlations for Early-Onset CAD Patients (n = 100)	
	Rs	*p*-Value
Age of patients (years)	−0.15	0.16
Past first MI (years)	−0.06	0.62
Age of hypertension diagnosis (years)	−0.23	0.06
Systolic BP * (mmHg)	−0.03	0.77
Diastolic BP (mmHg)	−0.08	0.48
MAP (mmHg)	−0.06	0.59
Heart rate (1/min)	−0.07	0.54
BMI (kg/m^2^)	0.22	0.035
Weight (kg)	0.17	0.11
WHR	0.12	0.29
Waist (cm)	0.19	0.07
Hip (cm)	0.15	0.17

* BP—blood pressure.

**Table 4:** Association between circulating TGF-β concentration (ng/mL) and clinical parameters of early-onset CAD patients.

Parameter	*p*-Value
Gender (% males)	0.70
Past MI	0.81
History of hypertension	0.25
Presence diabetes type 2	0.52
Past CABG	0.83
Past PTCA	0.57
Metabolic syndrome	0.23
Anti-platelet drugs (Aspirin)	0.84
Statins	0.44
Calcium channel blockers	0.45
Beta-blockers	0.14
Diuretics	0.07
ACEI	0.19
ARB	0.73
Anti-arrhythmic drugs	0.30
Digitalis	0.78
Nitrates	**0.00025**
Fibrates	0.63
Trimetazidine	0.34
NSAIDs *	0.19

* NSAIDs—nonsteroidal anti-inflammatory drugs.

**Table 5:** The correlations between circulating TGF-β concentration (ng/mL) and biochemistry parameters of early-onset CAD patients.

Parameters	Correlations	
	Rs	*p*-Value
TNF-α (pg/mL)	0.34	**0.0006**
IL6 (pg/mL)	0.08	0.44
VEGF (pg/mL)	0.22	0.03
sCD36 (µg/mL)	−0.25	0.02
PCSK9 (ng/mL)	0.11	0.29
E-selectin (ng/mL)	0.15	0.15
Glucose (mg/dL)	0.28	0.002
Platelets (G/L)	0.38	**0.0002**
Hemoglobin (g/dL)	0.19	0.07
WBC (G/L)	0.32	0.002
PTC	0.37	**0.0003**
hsCRP (mg/dL)	0.17	0.11
Triacylglycerol (mg/dL)	0.35	**0.0005**
LDL-cholesterol (mg/dL)	0.28	0.005
HDL-cholesterol (mg/dL)	−0.13	0.22
Total cholesterol (mg/dL)	0.23	0.02
Lp(a) (mg/dL)	−0.08	0.46
ApoB/ApoA1	0.26	0.01
ApoB (mg/dL)	0.32	0.002
ApoA (mg/dL)	−0.01	0.94

**Table 6:** Linear regression model with TGF-β concentration (ng/mL) as the dependent variable.

Variables	*p*-Value	β Coefficient (95%CI)
TNF-α	**0.00061**	+0.33 (+0.15–+0.52)
Platelets	0.0048	+0.27 (+0.08–+0.45)
Triacylglycerols	0.07	+0.17 (−0.02–+0.36)

## Data Availability

The Department of Biochemistry at Pomeranian Medical University holds data that supports the reported results. Those interested in obtaining this data should contact the corresponding author.
